# Sponging out a Headache

**Published:** 1891-09

**Authors:** 


### SPONGING OUT A HEADACHE.

In case of the ordinary nervous headache from which women suffer so much, says an authority, remove the dress waist, knot the hair high up on the head, out of the way, and, while leaning over the basin, place a sponge soaked in hot water, as hot as can be borne, on the back of the neck. Repeat this many times, also applying the sponge behind the ears, and, if the assertion of the writer is not a mistaken one, in many cases the strained muscles and nerves that have caused so much misery will be felt to relax and soothe themselves out deliciously ; and very frequently the pain promptly vanishes in consequence.

Every woman knows the aching face and neck generally brought home from a hard day's shopping, or from a long round of calls and afternoon teas. She regards with intense dissatisfaction the heavy lines drawn around her eyes and mouth by the long strain on the facial muscles, and when she must carry that worn countenance to some dinner party or evening's amusement it robs her of all the pleasure to be had in it. Cosmetics are not the cure, nor bromides or the many nerve sedatives to be had at the drug shop. Here again the sponge and hot water are advised by the writer quoted, bathing the face in water as hot as it can possibly be borne ; apply the sponge over and over again to the temples, throat and behind the ears, where most of the nerves and muscles of the head center, and then bathe the face in water running cold from the faucet. Color and smoothness of outline return to the face, an astonishing freshness and comfort results, and if followed by a nap of ten minutes all trace of fatigue vanishes.—*Health and Home.*

